# Indacaterol for Chronic Obstructive Pulmonary Disease: Systematic Review and Meta-Analysis

**DOI:** 10.1371/journal.pone.0070784

**Published:** 2013-08-14

**Authors:** Vincent C. H. Chung, Polly H. X. Ma, David S. C. Hui, Wilson W. S. Tam, Jin Ling Tang

**Affiliations:** 1 Jockey Club School of Public Health and Primary Care, The Chinese University of Hong Kong, Hong Kong, Hong Kong; 2 Shenzhen Municipal Key Laboratory for Health Risk Analysis, Shenzhen Research Institute of The Chinese University of Hong Kong, Shenzhen, Guangdong Province, China; 3 Department of Medicine and Therapeutics, The Chinese University of Hong Kong, Hong Kong, Hong Kong; University of Tübingen, Germany

## Abstract

**Background:**

Inhaled bronchodilators are the first-line therapy for COPD. Indacaterol is a novel addition to existing long-acting bronchodilators.

**Objectives:**

Systematic review of randomized controlled trials (RCT) on efficacy and safety of indacaterol as compared: 1) with placebo at different dosages, 2) with existing bronchodilators; (3) as add-on treatment to tiotropium.

**Methods:**

We searched 13 electronic databases, including MEDLINE, EMBASE and CENTRAL, and contacted the manufacturer for unpublished data. Primary outcome was mean FEV1 change at 12^th^ week, secondary outcomes included changes in SGRQ, TDI and BODE index at 6 months, exacerbation at 1 year, and worsening of symptoms.

**Results:**

Twelve eligible RCTs of moderate risk of bias included data from 10,977 patients. Compared to placebo, indacaterol improved FEV1 by a weighted mean difference (WMD) of 0.16 L (95%CI: 0.15, 0.18 L, p<0.001), homogeneously above the minimally important difference of 0.10 L. It offered clinically relevant improvement in all secondary outcomes except exacerbation. Magnitude of benefit did not differ significantly by dosage, but one treatment related death was reported at 300 ug. Efficacy of Indacaterol was similar to formoterol and salmeterol (FEV1 WMD = 0.04L, 95%CI: 0.01L, 0.07 L, p = 0.02); and tiotropium (FEV1 WMD = 0.01L, 95%CI: −0.01, 0.03L, p = 0.61). The use of indacaterol on top of tiotropium yielded additional improvement on FEV1 (WMD = 0.07 L, 95%CI: 0.05L, 0.10 L, p<0.001).

**Conclusion:**

Indacaterol is safe and beneficial for patients with COPD at dosage ≤150 ug. It may serve as a good alternative to existing bronchodilators, or as an add-on to tiotropium for unresponsive patients. Use of higher dosage requires further justification.

## Introduction

Chronic Obstructive Pulmonary Disease (COPD) is the fourth leading cause of mortality worldwide. It contributes to 27.2 age adjusted deaths per 100,000 US populations; and the figure reached 130.5 in China [1]. COPD is characterized by persistent airflow limitation that is progressive, and is associated with major co-morbidities. It has been estimated it will be the fifth leading cause of disability by 2020 [2]. Population aging directly raises the magnitude of economic burden caused by COPD, mainly due to higher cost incurred from acute care [3].

In the treatment of more symptomatic stable COPD patients, inhaled long acting β_2_ agonists or anticholinergic bronchodilators are superior to short-acting bronchodilators. Commonly prescribed β_2_ agonists include the twice daily formoterol or salmeterol, and for anticholinergic, the once daily tiotropium. For patients who do not respond well to monotherapy, combined use of β_2_ agonists and anticholinergic bronchodilators is suggested, although uncertainty remains in the appropriate timing for doing so [4], [5].

Indacaterol is a novel, once daily, inhaled ultra long acting β_2_ agonist approved by the European Medicines Agency (EMA) in 2009 at dosages of 150 and 300 ug. It has also gained approval from the US Food and Drug Administration (FDA) in 2011, but only at a lower dosage of 75 ug. The FDA has decided that the bronchodilation effects offered by 75 and 150 ug are similar, but higher dose is associated with respiratory related death [6]. The comparative efficacy and safety of the two EMA approved dosages (150 and 300 ug) has remained uncertain.

Beyond dosage, answers to three additional questions are needed for clarifying the role of indacaterol in treating stable COPD: What is the comparative effectiveness of indacaterol versus (i) existing β_2_ agonists of formoterol and salmeterol?; (ii) the anticholinergic tiotropium? (iii) Does the addition of indacaterol to tiotropium offer additional benefits to patients? We attempted to answer these questions by conducting a systematic review and meta-analysis of randomized controlled trials (RCTs) evaluating the efficacy and safety of indacaterol.

## Methods

### Data Sources and Search Strategy

To identify potentially relevant articles, we searched Cochrane Central Register of Controlled Trials (CENTRAL), MEDLINE, EMBASE and AMED using keywords related to COPD, indacaterol and RCTs. Sensitivity maximizing filters for identifying RCTs were applied in MEDLINE [7] and EMBASE [8]. The MEDLINE search strategy is listed in File S1. We also searched the following databases using the keyword “indacaterol”: Global Health, NHS Health Technology Assessment Database, Digital Dissertation Consortium, International Pharmaceutical Abstract and BIOSIS Preview. Furthermore, we searched the following trial registers of RCTs [9]:

CinicalTrial.gov (www.clinicaltrial.com),

Drugs@FDA (http://www.accessdata.fda.gov/scripts/cder/drugsatfda/index.cfm),

European Medicines Agency public assessment reports (EPAR, http://www.ema.europa.eu/ema),

Pharmaceuticals and medical devices agency of Japan (http://www.pmda.go.jp/english/service/approved.htmlhttp://www.pmda.go.jp/english/service/approved.html).

In all electronic searches, duration was the databases' inception till 30 Jan 2012. We applied no language restrictions. We also contacted authors of eligible studies for other existing publications via emails.

### Criteria for considering studies for this review

Two reviewers (VC and PM) independently screened electronically retrieved titles and abstracts, evaluated potentially relevant full texts, and determined study eligibility. We resolved disagreements on relevance by discussion and consensus adjudication. RCTs comparing indacaterol with control therapies (placebo or other drugs) for treating adults with stable COPD were eligible. The RCT must report change in FEV1 value with a minimum duration of 12 weeks, which was the primary outcome of this review. Secondary outcomes included exacerbation at or beyond 1 year, as well as changes in the following with a minimal duration of 6 months: Transition Dyspnoea Index (TDI), St George's Respiratory Questionnaire (SGRQ) scoring, and BODE index. Selection of endpoints was based on recommendations from the US FDA and the EMA [10]. Given the paucity of exacerbation data, we also evaluated worsening of COPD by the end of trial as a proxy.

### Data Extraction and Risk of Bias Assessment

Two authors (VC and PM) independently extracted data from included studies using a piloted data extraction form. We contacted corresponding authors and manufactures for unpublished or other additional data. Risks of bias of included studies were assessed using the Cochrane risk of bias tool [11] independently by the two reviewers. Discrepancies in data extraction and risk of bias assessment results were resolved by group consensus.

### Statistical analysis

All analyses were conducted using the Review Manager 5 software. Changes in continuous outcomes were expressed as weighted mean differences (WMD), while for dichotomous outcomes; relative risks (RR) were used. 95% confidence intervals (CI) were calculated for all estimates. We performed random effect meta-analysis separately for each outcome. *A priori*, we planned to conduct subgroup analyses according to dosage (≤150 ug, >150 ug), as well as comparators (placebo, formoterol and salmeterol; or tiotropium). For the primary outcome of FEV1 change, Egger's test was conducted to assess publication bias [12]. Tests for heterogeneity were performed with chi-squared testes, at a significance level of p = 0.1. I^2^ statistic was calculated to estimate total variation across studies. We regarded I^2^ <25% as an indicator of low heterogeneity level, 25–50% as moderate level, and higher than 50% as high level [13]. Heterogeneity was explored with sensitivity analysis.

To aid interpretation, synthesized estimates were compared against the minimally important difference (MID) values for each of the outcomes: 0.10 L for FEV1 [14] 1 unit for TID [15] and 4 points for SGRQ scoring [16]. For BODE index, an improvement equal or larger than 1.19 fold was considered to be clinically important [17]. We also attempted to summarize the following adverse outcomes: death related to treatment, any reported adverse events, serious adverse events, upper respiratory tract infection (URTI), nasopharyngitis and cough.

## Results

### Literature search

**Figure 1:**
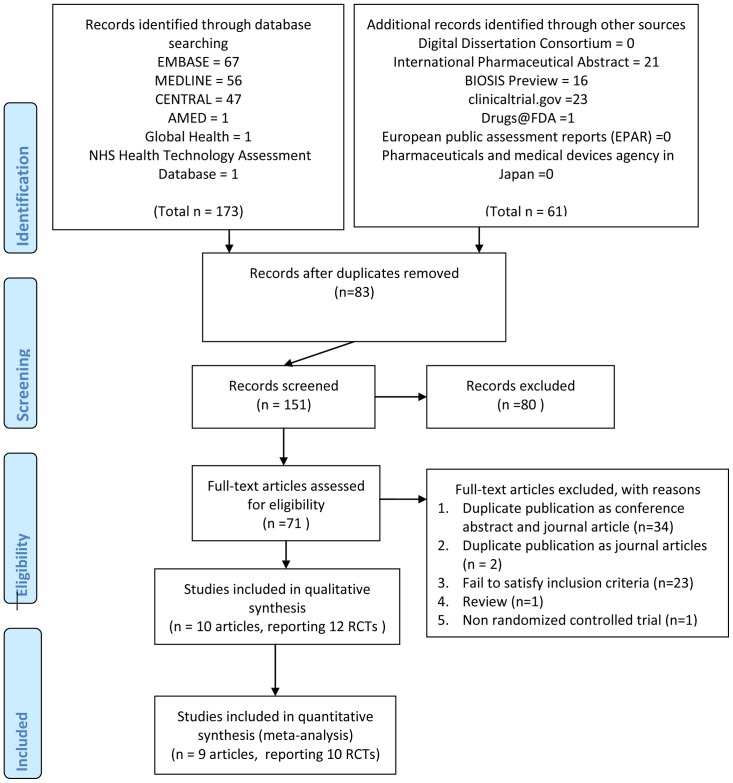
The flow of literature search.

We identified a total of 234 citations from all searches and excluded 83 duplicates. After screening titles and abstracts, we retrieved 71 full texts for further assessment. Of these, 38 were excluded for the following reasons: duplicate publications as conference abstracts and journal articles (n = 34), duplication publications as journal articles (n = 2), did not report specified primary endpoint (n = 23), review (n = 1), and non RCTs (n = 1). Nine full texts and one abstracts (based on their latest publication form), which reported 12 RCTs, were eligible for inclusion. The flow of literature search is illustrated in Figure 1.

### Characteristics of included studies

Characteristics of included trials are summarized in Table 1. These 12 RCTs recruited a total of 10,977 COPD patients (mean = 1,097; SD = 701.1; median = 1061.5; range = 186–2271). In all studies, COPD diagnosis was made according to the Global Initiative for Chronic Obstructive Lung Disease (GOLD) criteria of having a post-bronchodilator FEV_1_<80% of the predicted value, as well as a ratio of FEV1 to forced vital capacity (FVC) <70% [5]. Overall, the majority of the included patients were of moderate to severe severity.

**Table 1:** Characteristics of included studies.

Source	Trial length	Total no. of participants	Intervention	Age (SD)	Sex Male/female %	Duration of COPD years (SD)	Ex-smoker/smoker	Smoking history, pack-years (SD)	ICS use %	Baseline FEV_1_, % predicted, (SD)	Baseline FEV_1_/FVC (SD)	%FEV_1_ reversibility (SD)
Korn et al, 2011	12 weeks	1121	Indacaterol 150 ug once daily	62.4 (8.86)	66.2/33.8	6.8 (5.75)	54.9/45.1	44.6 (23.37)	45.8	52.1 (12.03)	51.1 (9.08)	14.4 (13.29)
			Salmeterol 50 ug twice daily	63.2 (8.69)	73.8/26.2	7.4 (5.88)	56.0/44.0	45.0 (24.27)	46.1	51.5 (12.60)	50.2 (9.95)	14.4 (13.62)
Kerwin	12	641	Study 1									
et al, 2011	weeks		Indacaterol 75 ug once daily	64 (8.3)	55/45	7 (6.3)	56/44	53 (26.8)	43.0	54 (12.8)	53 (9.5)	15 (12.7)
			Placebo	64 (9.4)	54/46	7 (6.4)	56/44	51 (24.8)	48.0	53 (13.4)	52 (10.6)	17 (13.9)
			Study 2									
			Indacaterol 75 ug once daily	61 (9.8)	52/48	7 (6.1)	42/58	52 (28.1)	40.0	56 (12.8)	52 (10.3)	18 (16.7)
			Placebo	62 (9.9)	56/44	7 (6.1)	40/60	52 (28.4)	35.0	54 (13.6)	53 (9.9)	16 (13.9)
Feldman et al, 2010	12 weeks	416	Indacaterol 150 ug once daily	62.9 (9.89)	51.2/48.8	6.6 (6.86)	48.8/51.2	53.5 (26.84)	28.9	54.4 (13.38)	53.5 (9.84)	16.4 (17.31)
			Placebo	63.2 (9.62)	53.7/46.3	7.3 (5.64)	47.3/52.7	60.5 (54.12)	34.1	55.8 (14.08)	53.5 (10.36)	16.6 (19.44)
Buhl et al, 2011	12 weeks	1598	Indacaterol 150 ug once daily	63.6 (8.60)	70/30	7.0 (6.01)	55/45	43.2 (20.87)	54.0	54.6 (12.80)	51.0 (9.38)	14.1 (12.63)
			Tiotropium 18 ug once daily	63.4 (8.29)	67/33	7.0 (6.32)	56/44	41.8 (19.81)	56.0	54.3 (12.81)	51.2 (9.42)	13.7 (13.44)
Kinoshita et al, 2012	12 weeks	347	Indacaterol 150 ug once daily	66.4 (8.75)	96.5/3.5	4.2 (3.74)	64.9/35.1	51.7 (29.21)	21.9	55.2 (12.77)	50.3 (10.55)	14.7 (12.88)
			Indacaterol 300 ug once daily	67.1 (7.67)	97.4/2.6	3.4 (3.44)	66.4/33.6	54.0 (28.56)	21.6	53.7 (12.67)	48.7 (9.61)	15.3 (14.86)
			Placebo	66.5 (8.74)	95.7/4.3	3.9 (3.97)	72.6/27.4	49.7 (27.96)	29.1	52.3 (11.98)	47.7 (10.41)	15.3 (12.58)
Mahler et al, 2012	12 weeks	2276	Study 1									
			Indacaterol 150 ug + tiotropium 18 ug once daily	64.0 (9.07)	70/30	7.1(6.12)	60/40	47.2 (25.86)	52	48.3 (9.70)	46.4 (9.74)	18.5 (15.68)
			Tiotropium 18 ug + Placebo once daily	63.4 (9.22)	67/33	6.6(6.45)	64/36	47.2 (26.58)	52	48.9 (11.46)	45.8 (10.00)	16.6 (14.10)
			Study 2									
			Indacaterol 150 ug + tiotropium 18 ug once daily	63.1 (8.83)	63/37	7.3(6.48)	62/38	46.2 (25.52)	57	48.6 (9.74)	47.0 (10.21)	16.4 (15.32)
			Tiotropium 18 ug + Placebo once daily	62.8 (8.98	68/32	7.7(6.26)	57/43	46.3 (24.64)	51	48.6 (9.76)	47.2 (9.53)	16.5 (15.20)
Kornmann et al, 2010	26 weeks	1002	Indacaterol 150 ug once daily	63 (8.7)	72/28	6.5 (5.7)	54/46	40 (17.0)	45.0	54.0 (14.0)	50.0 (10.0)	12 (15.3)
			Salmeterol 50 ug twice daily	63 (9.2)	75/25	6.4 (5.7)	54/46	40 (16.7)	46.0	53.0 (13.6)	50.0 (10.0)	11 (13.9)
			Placebo	64 (8.6)	77/23	6.6 (5.8)	55/45	41 (18.9)	40.0	53.0 (14.2)	50.0 (11.0)	13 (16.4)
Donohue et al, 2010	26 weeks	1665	Indacaterol 150 ug once daily	63.4 (9.40)	62.3/37.7	*	*	48.3 (23.4)	38.2	56.1 (14.5)	53.0 (10.0)	15.6 (15.4)
			Indacaterol 300 ug once daily	63.3 (9.32)	62.2/37.8	*	*	50.8 (27.7)	37.3	56.3 (14.5)	52.6 (10.1)	15.2 (15.4)
			Tiotropium 18 ug once daily	64.0 (8.77)	64.8/35.2	*	*	50.0 (25.1)	34.9	53.9 (15.6)	52.7 (10.1)	15.6 (17.6)
			Placebo	63.6 (8.92)	61.0/39.0	*	*	49.7 (23.9)	39.5	56.1 (14.3)	53.4 (10.1)	15.5 (18.0)
Dahl et al, 2010	52 weeks	1728	Indacaterol 300 ug once daily	64.0 (57.0, 71.0)┼	80.3/19.7	*	*	40.0 (30.0,53.0)┼	55.6	51.5 (42.2, 62.9)┼	50.7 (43.5, 59.5)┼	9.8 (3.0, 18.3)┼
			Indacaterol 600 ug once daily	63.0 (57.0, 69.0) ┼	76.9/23.1	*	*	40.0 (30.0, 58.0)┼	53.2	50.8 (41.2, 60.2) ┼	51.1 (43.8, 59.1)┼	10.9 (3.8, 20.6)┼
			Formoterol 12 ug twice daily	64.0 (58.0, 69.0)┼	80.2/19.8	*	*	40.0 (30.0, 50.0)┼	50.9	52.5 (41.2, 63.1)┼	51.2 (43.5, 59.0)┼	10.1 (3.4, 18.3)┼
			Placebo	63.0 (57.5, 69.0)┼	81.5/18.5	*	*	43.0 (31.0, 53.5)┼	51.9	52.0 (41.9, 63.6)┼	52.0 (44.1, 60.5) ┼	10.8 (4.7, 19.1)┼
To et al, 2011	52 weeks	186	Indacaterol 300 ug once daily	*	*	*	*	*	*	*	*	*
			Salmeterol 50 ug twice daily	*	*	*	*	*	*	*	*	*

Data are presented as % or mean ± SD, unless otherwise stated.

┼ Data are presented as median (upper and lower quartiles).

* Details not reported.

Abbreviations: COPD: chronic obstructive pulmonary disease; FEV_1_: forced expiratory volume in 1 s; FVC: forced vital capacity; ICS: inhaled corticosteroids.

The mean indacaterol dose was 225 ug/d (range, 75–600 ug/d). Four studies were placebo-controlled [18]–[20]. Two studies compared indacaterol with salmeterol [21], [22], and one had a three arms design including indacaterol, salmeterol and placebo [23]. One compared indacaterol with tiotropium [24], whereas one had a four-arm design of placebo, tiotropium and two dosages of indacaterol [25]. Two evaluated the combined effect of indacaterol and tiotropium, compared to tiotropium alone [26]. Finally, one had a four-arm design of placebo, formoterol and two dosages of indacaterol [27].

### Risk of bias

Risk of bias amongst included studies was mediocre overall (Table 2), with poor reporting on methodological details. None of them provided details on methods for generating random sequence. Implementation of allocation concealment was described in eight RCTs, and details were unclear in the remaining four. Seven RCTs blinded both patients and investigators but two did not, and three did not provide sufficient information for judgment. Three RCTs reported blinding of assessors but not the remaining nine. However, we consider the risk of bias incurred from this to be low because the impact of lack of blinding on the measurement of FEV1, an objective primary outcome, is rather low [28]. The drop-out rates ranged from 6.1% to 26.0%, with a mean (SD) of 14.1% (6.72%) and a median of 12.3%.

**Table 2:** Risk of bias amongst included studies.

Source	Sequence generation	Allocation Concealment	Blinding of participants and researchers	Blinding of outcome assessment#	Incomplete Outcome Data Addressed
Korn et al, 2011	Unclear risk, sequence generation method not stated.	Low risk. Automated interactive voice response system used.	Low risk. Blinding was maintained by providing placebo matching for both treatments	Low risk. Blinding of assessors not mentioned but its impact maybe low since FEV1 is an objective outcome measure	Low risk. Proportion of drop-out amongst study groups differ by ≤10%. 89/1121 patients dropped out, 49 in Indacaterol group and 40 in control group. Drop-out rate: 7.94%
Kerwin et al, 2011	Unclear risk, sequence generation method not stated.	Low risk. Automated interactive voice response & web system	Low risk. Double-blinding on patients and investigating staffs.	Low risk. Assessors were blinded from randomization to study completion.	Low risk. Proportion of drop-out amongst study groups differ by ≤10%. 49/323 patients in study 1 dropped out, 19 in Indacaterol group and 30 in control group. Drop-out rate of study 1: 15.17%. 28/318 patients in study 2 dropped out, 11 in Indacaterol group and 17 in control group. Drop-out rate of study 2: 8.81%
Feldman et al, 2010	Unclear risk, sequence generation method not stated.	Unclear, details not stated	Low risk. Patients and investigators were blinded from the time of randomization to database lock	Low risk. Clinical staffs performing assessment were blinded from the time of randomization to database lock	Low risk. Proportion of drop-out amongst study groups differ by ≤10%. 52/416 patients dropped out, 25 in Indacaterol group and 27 in control group.Drop-out rate:12.5%
Buhl et al, 2011	Unclear risk, sequence generation method not stated.	Low risk. The assigned treatment was dispensed to patients by a third party who was not otherwise involved in the study	Low risk. Patients were blinded to treatment assignment. Investigators were blinded and did not observe the actual treatment patients took at clinic visits	Low risk. Study staff performing the assessments were blinded and did not observe the actual treatment patients took at clinic visits	Low risk. Proportion of drop-out amongst study groups differ by ≤10%. 124/1598 patients dropped out, 60 in Indacaterol group and 64 in control group. Drop-out rate:7.76%
Kinoshita et al, 2012	Unclear risk, sequence generation method not stated.	Unclear, details not stated.	Unclear risk The study did not mention blinding of participants and researchers	Low risk#. Blinding of assessors not mentioned but its impact maybe low since FEV1 is an objective outcome measure	Low risk. Proportion of drop out amongst study groups differ by ≤10%. 39/347 patients dropped out, 20 in Indacaterol group and 19 in control group Drop-out rate:11.2%
Mahler et al, 2012	Unclear risk, sequence generation method not stated.	Low risk. Automated interactive voice response system used. The authors stated that “patients and staff at participating centers were unaware of treatment assignment”.	Low risk. Blinding of researchers and patients were achieved by using placebo delivered via a indacaterol inhaler.	Low risk#. Blinding of assessors not mentioned but its impact maybe low since FEV1 is an objective outcome measure	Low risk. Proportion of drop out amongst study groups differ by ≤10%. 74/1134 patients in study 1 dropped out. Drop-out rate of study 1: 6.50%. 66/1142 patients in study 2 dropped out. Drop-out rate of study 2: 5.80%
Kornmann et al, 2010	Unclear risk, sequence generation method not stated.	Unclear, details not stated.	Unclear risk. The study did not state explicitly on the blinding of participants and researchers, although it was mentioned that “placebos matching both active treatments were used to maintain blinding”.	Low risk#. Blinding of assessors not mentioned but its impact maybe low since FEV1 is an objective outcome measure	Low risk. Proportion of drop out amongst study groups differ by ≤10%. 164/1002 patients dropped-out, 44 in Indacaterol group, 50 in Salmeterol group, and 70 in placebo group. Drop-out rate:16.4%
Donohue et al, 2010	Unclear risk, sequence generation method not stated.	Low risk. Automated interactive voice response system used.	High risk. The study failed to blind tiotropium treatment	Low risk#. Blinding of assessors not mentioned but its impact maybe low since FEV1 is an objective outcome measure	High risk. Proportion of drop-out amongst study groups differ by >10%. 392/1665 patients dropped-out, 172 in the Indacaterol group, 89 in Tiotropium group, and 131 in placebo group. Drop-out rate:23.5%
Dahl et al, 2010	Unclear risk, sequence generation method not stated.	Low risk. Automated interactive voice response system used.	Unclear risk. The study did not mention blinding of participants and researchers	Low risk#. Blinding of assessors not mentioned but its impact maybe low since FEV1 is an objective outcome measure	Low risk. Proportion of drop-out amongst study groups differ by ≤10%. 450/1728 patients dropped-out, 201 in Indacaterol group, 112 in Formoterol group, and 137 in placebo group. Drop-out rate:26.0%
To et al, 2011	Unclear risk, sequence generation method not stated.	Unclear, details not stated.	High risk. Both treatments were administered open label	Low risk#. Blinding of assessors not mentioned but its impact maybe low since FEV1 is an objective outcome measure	Unclear risk. Insufficient information to permit judgment 33/186 patients dropped out. Drop out rate:17.7%

#Assessed based in the primary outcome of this review, FEV1 measurement. We assumed that the impact of assessor blinding on FEV1 measurement to be minimal.

### Indacaterol versus Placebo

#### Changes in FEV1

**Figure 2:**
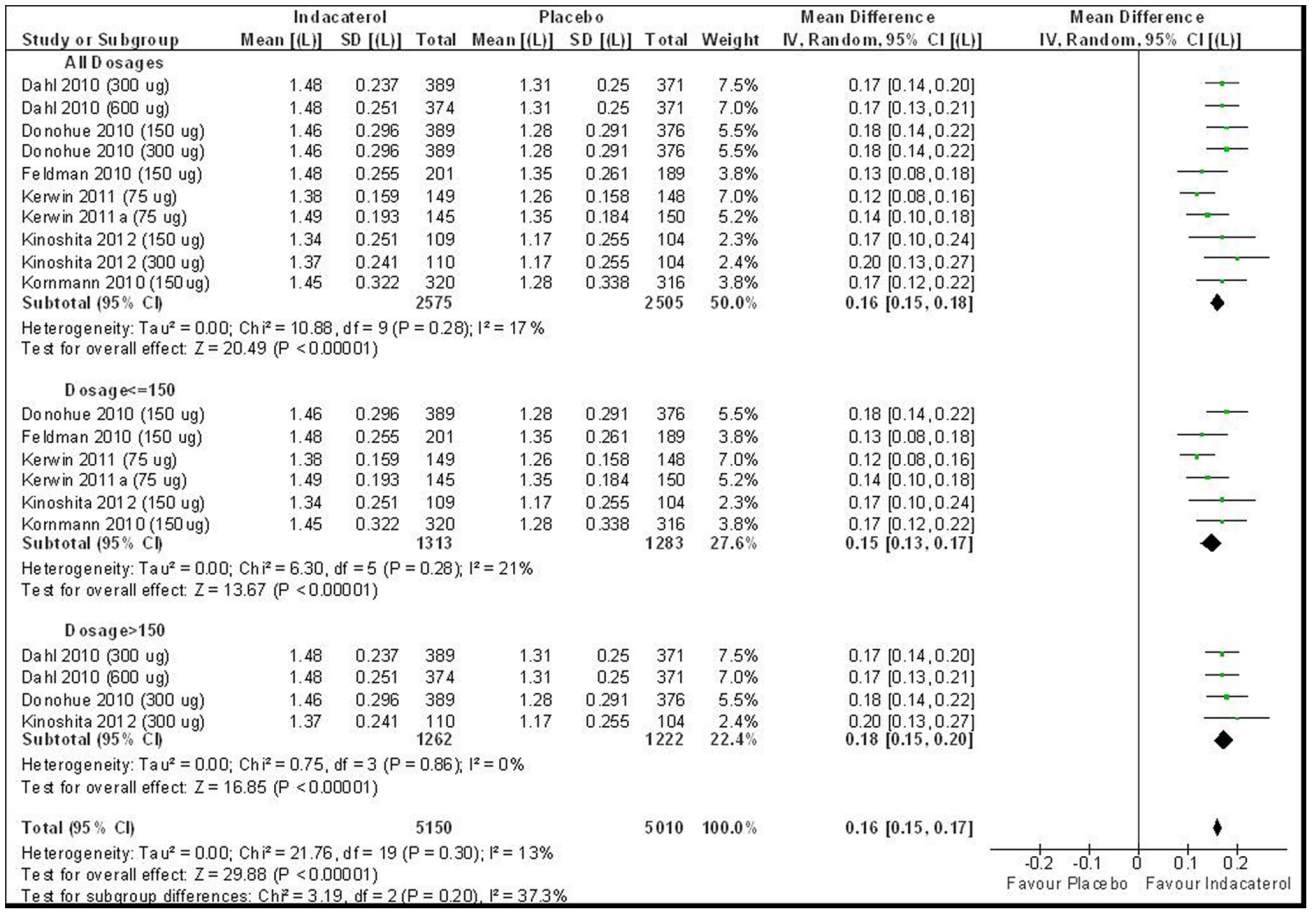
Indacaterol versus Placebo on FEV1 at 12 weeks.

In this comparison, a total of 10 RCTs (n = 5,080) reported adjusted FEV1 change at 12 weeks. Pooled results demonstrated homogeneous superiority of Indacaterol above the MID value of 0.10 L, with a weighted mean difference (WMD) of 0.16 L (95%CI: 0.15 L to 0.18 L, I^2^ = 17%, Figure 2). Egger's test showed no signs of publication bias (z = 0.40, p = 0.69).

In a subgroup analysis (Figure 2) limiting to six trials using ≤150 ug (n = 2,596) and four trials >150 ug (n = 2,484), pooled results remained to be higher than the MID value. For dosage ≤150 ug, the WMD is 0.15 L (95%CI: 0.13 L to 0.17 L, I^2^ = 21%); while WMD for >150 ug is 0.18 L (95%CI: 0.15 L to 0.20 L, I^2^ = 0%). There was no statistically significant difference between the two pooled estimates (p = 0.066), and there was no significant correlation between dosage and mean difference in FEV1 (r = 0.49, p = 0.16).

#### Changes in SGRQ, TDI, BODE Index, exacerbation rate and worsening of symptoms

In one trial, both Indacaterol 300 ug and 600 ug were found to be superior to placebo in improving SGRQ scoring above the MID value of 4 at 52^nd^ week, with a mean difference of −4.7 and −4.8 respectively [27]. Similar change at 26^th^ week was reported by one trial using 150 ug [23], but another trial testing 150 ug and 300 ug did not find clinically relevant improvements at the same time point [25]. At both 150 and 300 ug, indacaterol improved TDI at 26^th^ and 52^nd^ weeks at its MID value of 1 unit [23], [25], [27]. In one trial, indacaterol 300 ug and 600 ug respectively improved BODE index by 1.23 and 1.21 folds^27^, which were considered to be clinically relevant at 52^nd^ week (Table 3).

**Table 3:** Efficacy Results of Indacaterol by Comparison Type for SGRQ, TDI and BODE index.

Study (Publication Year)	Comparison	Indacaterol Group (n)	Adjusted mean (Standard error)	Control Group (n)	Adjusted mean (Standard error)	Mean difference (Standard error)
**Outcome: SGRQ at 26^th^ week**						
Kornmann et al.(2010)	Indacaterol 150ug vs. Placebo	299	36.8 (1.04)	274	41.8 (1.07)	−5.0 (1.49)
	Indacaterol 150ug vs. Salmeterol 50ug	299	36.8 (1.04)	292	37.8 (1.05)	−1.0 (1.48)
Donohue et al.(2010)	Indacaterol 150ug vs. Placebo	346	37.1 (0.78)	319	40.4 (0.79)	−3.3 (1.11)
	Indacaterol 300ug vs. Placebo	360	38.0 (0.76)	319	40.4 (0.79)	−2.4 (1.10)
	Indacaterol 150ug vs. Tiotropium 18ug	346	37.1 (0.78)	357	39.4 (0.76)	−2.3 (1.09)
	Indacaterol 300ug vs. Tiotropium 18ug	360	38.0 (0.76)	357	39.4 (0.76)	−1.4 (1.07)
	Indacaterol 150ug vs. Indacaterol 300ug	346	37.1 (0.78)	360	38.0 (0.76)	−0.9 (1.09)
**Outcome: SGRQ at 52^nd^ week**						
Dahl et al.(2010)	Indacaterol 300ug vs. Placebo	322	36.5 (0.82)	280	41.3 (0.87)	−4.8 (1.20)
	Indacaterol 300ug vs. Formoterol 12ug	322	36.5 (0.82)	302	37.3 (0.84)	−0.8 (1.17)
	Indacatero 600ug vs. Placebo	305	36.6 (0.83)	280	41.3 (0.87)	−4.7 (1.20)
	Indacaterol 600ug vs. Formoterol 12ug	305	36.6 (0.83)	302	37.3 (0.84)	−0.7 (1.18)
	Indacaterol 300ug vs. Indacaterol 600ug	322	36.5 (0.82)	305	36.6 (0.83)	−0.1 (1.17)
**Outcome: TDI at 24^th^ week**						
To et al.(2011)	Indacaterol 300ug vs Salmeterol 50ug	118	0.60 (0.222)	56	0.12 (0.295)	0.48 (0.369)
**Outcome: TDI at 26^th^ week**						
Kornman et al (2010)	Indacaterol 150ug vs. Placebo	297	2.03 (0.294)	272	1.04 (0.300)	0.99 (0.420)
Donohue et al. (2010)	Indacaterol 150ug vs. Salmeterol 50ug	297	2.03 (0.294)	289	2.02 (0.295)	0.01 (0.416)
	Indacaterol 150ug vs. Placebo	343	2.41 (0.230)	309	1.40 (0.234)	1.01 (0.328)
	Indacaterol 300ug vs. Placebo	353	2.58 (0.226)	309	1.40 (0.234)	1.18 (0.325)
	Indacaterol 150ug vs. Tiotropium 18ug	343	2.41 (0.230)	349	2.27 (0.228)	0.14 (0.324)
	Indacaterol 300ug vs. Tiotropium 18ug	353	2.58 (0.226)	349	2.27 (0.228)	0.31 (0.321)
	Indacaterol 150ug vs. Indacaterol 300ug	343	2.41 (0.230)	353	2.58 (0.226)	−0.17 (0.322)
**Outcome: TDI at 52^nd^ week**						
Dahl et al (2010)	Indacaterol 300ug vs. Placebo	317	2.57 (0.219)	280	1.57 (0.230)	1.00 (0.318)
	Indacaterol 300ug vs. Formoterol 12ug	317	2.57 (0.219)	300	2.28 (0.223)	0.29 (0.313)
	Indacatero 600ug vs. Placebo	299	2.55 (0.222)	280	1.57 (0.230)	0.98 (0.320)
	Indacaterol 600ug vs. Formoterol 12ug	299	2.55 (0.222)	300	2.28 (0.223)	0.27 (0.315)
	Indacaterol 300ug vs. Indacaterol 600ug	317	2.57 (0.219)	299	2.55 (0.222)	0.02 (0.312)
To, et al (2011)	Indacaterol 300ug vs Salmeterol 50ug	105	0.76 (0.227)	50	0.57 (0.289)	0.19 (0.367)
**Outcome: BODE at 52^nd^ week**						
Dahl et al (2010)	Indacaterol 300ug vs. Placebo	304	2.35 (0.071)	261	2.90 (0.076)	−0.55 (0.104)?
	Indacaterol 300ug vs. Formoterol 12ug	304	2.35 (0.071)	292	2.36 (0.072)	−0.01 (0.101);
	Indacatero 600ug vs. Placebo	292	2.40 (0.072)	261	2.90 (0.076)	−0.50 (0.105)#
	Indacaterol 600ug vs. Formoterol 12ug	292	2.40 (0.072)	292	2.36 (0.072)	0.04 (0.102)
	Indacaterol 300ug vs. Indacaterol 600ug	304	2.35 (0.071)	292	2.40 (0.072)	−0.05 (0.101)

? representing a 1.23 fold increase; # representing a 1.21 fold increase.

For the prevention of exacerbation at 1 year, one trial reported that 600 ug, but not 300 ug, fared better than placebo (RR for 600 ug = 0.74; 95%CI: 0.56 to 0.97; RR for 300 ug = 0.82; 95% CI: 0.63 to 1.06) [27]. A total of seven RCTs (n = 5,580) reported worsening of COPD symptoms (dyspnea, cough, sputum purulence/volume, or wheeze) at the end of the study [18]–[20], [23], [25], [27]. Pooled results demonstrated marginal superiority of Indacaterol in preventing worsening of symptoms, with a RR of 0.85 (95%CI: 0.77 to 0.94, I^2^ = 0%). In subgroup analyses limiting to six trials (n = 2,787) using ≤150 ug, the RR was 0.84 (95%CI: 0.70 to 1.00, I^2^ = 0%), while in four trials (n = 2,793) using >150 ug, the RR was 0.85 (95%CI: 0.75 to 0.96, I^2^ = 0%). There was no statistically significant difference between the two pooled estimates (p value of In RR  = 0.91).

### Comparison of Indacaterol at different dosages

One trial [25] directly compared the efficacy of indacaterol at 150 ug and 300 ug. The result showed no clinically relevant difference in FEV1 at both 12^th^ week (0.00 L, SE  = 0.02 L) and at 26^th^ week (0.03 L, SE  = 0.02 L). Differences in SGRQ and TDI were also below MID threshold. Another trial^27^ comparing 300 ug and 600 ug also reported similarities in FEV1 improvements at both 12^th^ week (0.00 L, SE  = 0.02 L) and 52^nd^ week (0.00 L, SE  = 0.02 L). Differences in SGRQ, TDI and BODE were also below MID thresholds (Table 3).

### Indacaterol versus other long acting β_2_ agonist bronchodilators

#### Changes in FEV1

**Figure 3:**
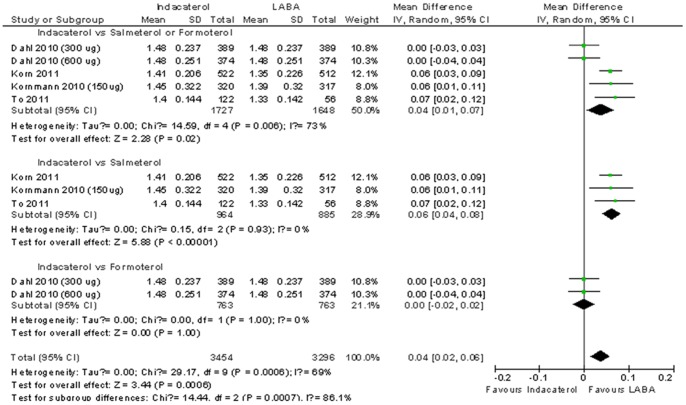
Indacaterol versus other long acting β_2_ agonist bronchodilators on FEV1 at 12 weeks.

In this comparison, a total of 4 RCTs (n = 3,375) reported adjusted FEV1 change at 12 weeks. One trial used formoterol as comparator [27] and the other three evaluated salmeterol [21]–[23]. Pooled results showed superiority of Indacaterol over the two existing long acting β_2_ agonist bronchodilators (WMD  = 0.04 L, 95%CI: 0.01 L to 0.07 L, Figure 3). Heterogeneity existed in this pooling (I^2^ = 73%) and accordingly we conducted subgroup analyses according to comparator. Compared to salmeterol, combined result favors Indacaterol homogeneously (WMD  = 0.06 L, 95%CI: 0.04 L to 0.08 L, I^2^ = 0%), but the magnitude was below MID threshold. No statistically significant difference between indacaterol and formoterol was observed (WMD  = 0.04 L, 95%CI: –0.02 L to 0.02 L, I^2^ = 0%) (Figure 3).

At longer term, indacaterol appeared to be superior to salmeterol but the magnitudes of differences were clinically insignificant. One trial reported mean differences in FEV1 of 0.06 L (SE  = 0.03 L) and 0.08 L (SE  = 0.03 L), respectively at 24^th^ and 52^nd^ weeks [22]. Another trial using 26^th^ weeks FEV1 endpoint showed a mean difference of 0.07 L (SE  = 0.03 L) [23]. Compared to formoterol, one trial reported a mean difference of 0.00 L (SE  = 0.02 L) at 52^nd^ weeks, for both indacaterol dosages of 300 ug and 600 ug [27].

#### Changes in SGRQ, TDI, BODE and exacerbations

No clinically relevant difference between salmeterol and indacaterol was observed in the outcomes of SGRQ [23] and TDI [22], [23]. Similarly, the differences between formoterol and indacaterol on the outcomes of SGRQ, TDI and BODE index were below MID threshold (Table 3). No trial under this comparison reported exacerbation rate at one year.

### Indacaterol versus Tiotropium

**Figure 4:**
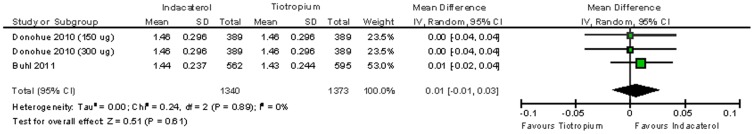
Indacaterol versus Tiotropium on FEV1 at 12 weeks.

In this comparison, two RCTs (n = 2,713) reported adjusted FEV1 change at 12 weeks [25], [27]. Pooling did not show a statistically significant difference between the two drugs (WMD  = 0.01 L, 95%CI = −0.01 L to 0.03 L, I^2^ = 0%, Figure 4). At 26 weeks, one trial reported that the mean FEV1 difference between indacaterol and tiotropium were 0.04 L (SE  = 0.02 L) and 0.01 L (SE  = 0.02 L), respectively at 300 ug and 150 ug^25^. Both estimates were below MID threshold, and similar efficacies between the two drugs were also observed in the outcomes of SGRQ and TDI (Table 3).

### Indacaterol plus Tiotropium versus Tiotropium plus placebo

**Figure 5:**
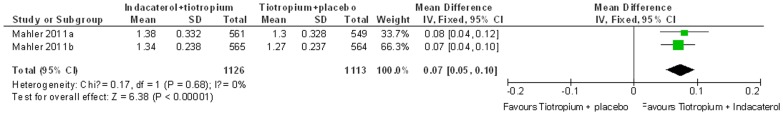
Indacaterol plus Tiotropium versus Tiotropium plus placebo on FEV1 at 12 weeks.

In this comparison, a total of 2 RCTs (n = 2,239) reported adjusted FEV1 change at 12 weeks [26]. The pooled WMD was 0.07 L (95%CI: 0.05 L to 0.10 L, I^2^ = 0%, Figure 5). This demonstrated the additional benefit from Indacaterol on top of tiotropium treatment, with the upper 95% CI of the effect size approaching MID threshold. None of the pre-specified secondary outcomes were reported in the trial.

### Adverse events

Amongst all included RCTs, one death was found to be related to the use of Indacaterol at 300 ug [27]. Indacaterol users were significantly more likely to experience nasopharyngitis, compared to those who used placebo (RR  = 1.22, 95%CI: 1.01 to 1.47, I^2^ = 15%). In subgroup analysis, this result was statistically significant only at dosage >150 ug (RR >150 ug  = 1.27, 95%CI: 1.04 to 1.54, I^2^ = 0%; RR ≤150 ug  = 1.24, 95%CI: 0.80 to 1.91). Nevertheless, the difference between the two effect sizes was statistically insignificant (p value of In RR  = 0.92). Occurrence of the following adverse events did not differ significantly between indacaterol and placebo, in both overall and subgroup analyses: any reported adverse events, serious adverse events, URTI, and cough (Table 4).

**Table 4:** Meta-analysis on adverse events: Indacaterol versus Placebo.

Events	No. of studies	Event/Total	Event/Total	Combined Effect		Heterogeneity
				RR (95% CI)	P -value	I^2^ (%)
			Any dose			
Any reported adverse events	10	1673/2787	1595/2793	1.05 (1.00, 1.10)	0.05	19%
Serious adverse events	8	120/1925	132/1929	0.92 (0.73, 1.17)	0.52	0%
Upper respiratory tract infection	7	148/2254	156/2269	0.96 (0.73, 1.27)	0.79	31%
Nasopharyngitis	9	285/2576	232/2588	1.22 (1.01, 1.47)	0.04	15%
Cough	10	178/2787	152/2793	1.16 (0.91, 1.46)	0.22	14%
			Indacaterol ≤150 ug			
Any reported adverse events	6	757/1393	726/1394	1.04(0.97–1.12)	0.23	0%
Serious adverse events	6	86/1393	91/1394	0.96 (0.72–1.27)	0.76	0%
Upper respiratory tract infection	3	65/860	48/870	1.35(0.94–1.95)	0.10	0%
Nasopharyngitis	5	85/1182	73/1189	1.24(0.80–1.91)	0.33	37%
Cough	6	78/1393	71/1394	1.09(0.74–1.61)	0.65	24%
			Indacaterol >150 ug			
Any adverse events	4	916/1394	869/1399	1.05(0.96–1.15)	0.32	56%
Serious adverse events	2	34/532	41/535	0.75(0.34–1.66)	0.47	30%
Upper respiratory tract infection	4	83/1394	108/1399	0.77(0.59–1.02)	0.07	0%
Nasopharyngitis	4	200/1394	159/1399	1.27(1.04–1.54)	0.02	0%
Cough	4	100/1394	81/1399	1.23(0.90–1.68)	0.20	16%

Notes*: RR:* risk ratio; *95%CI:* 95%confidence interval.

## Discussion

### Summary of main results

This systematic review has shown that indacaterol was effective in improving FEV1, SGRQ, TDI and BODE amongst patients with moderate to severe stable COPD. For these outcomes, sizes of benefit were above MID threshold and they did not vary by dosage. Indacaterol prevented worsening of symptoms but the size of benefit was marginal. It did not outperform placebo in preventing exacerbation at 1 year, even at its maximum approved dose of 300 ug. It had an acceptable safety profile except for slightly higher tendency in causing nasopharyngitis. Amongst 906 patients using a dose of 300 ug, one death was reported to be related to this treatment.

The efficacy of Indacaterol appears to be on par with all three long-acting bronchodilators recommended by the GOLD document: salmeterol, formoterol and tiopropium. Indacaterol was more effective than salmeterol in increasing FEV1, but the difference was too small to be clinically relevant. They were also similarly effective in improving SGRQ and TDI. For indacaterol and formoterol, they were no significant differences in their abilities in improving FEV1, SGRQ, TDI and BODE index. Furthermore, similar efficacies between indacaterol and tiotropium were observed in the outcomes of FEV1, SGRQ and TDI. The addition of indacaterol on top of tiotropium yielded extra improvement in FEV1, with an effect size approaching MID.

### Overall completeness and applicability of evidence

While indacaterol represents a new option for treating stable COPD, the case for using a dosage >150 ug is weak as this does not provide patients with additional benefits. More importantly, one death was reported at 300 ug, the maximum dose approved by the EMA. However, results from head to head equivalence trials comparing 75 and 150 ug are needed to draw a firm conclusion on their comparative effectiveness.

The once daily Indacaterol shares similar efficacy profile with its twice daily β_2_ agonist counterparts, formoterol and salmeterol. Clinicians may prefer indacaterol as the β_2_ agonist bronchodilator of choice, as once daily administration may enhance patient adherence, [29] and subsequently reduce risk of death and hospitalization [30]. Efficacy of indacaterol is similar to that of once daily anticholinergic bronchodilator, tiotropium. For patients who are intolerant to the anticholingeric side effect of tiotropium (e.g. dry mouth),[31] indacaterol may be an alternative.

Nevertheless, since exacerbation strongly predicts rapid decline in health status and death [32], uncertainty on indacaterol's efficacy in preventing exacerbations has cast doubt on this choice. Currently, no included trial reported the efficacy of indacaterol in preventing exacerbation beyond 1 year at dosage <300 ug. Results from future trials addressing this outstanding question are needed for guiding the choice between indacaterol and tiotropium. A recent trial has indicated the superiority of tiotropium over salmeterol in preventing exacerbations amongst patients with moderate to very severe COPD [33]. Hence, tiotropium may remain to be the preferred drug for patients prone to exacerbations until further evidence is available.

Addition of indacaterol seems to provide extra benefit on FEV1 amongst patients who are already using tiotropium. The combination has led to an additional FEV1 increment of 0.07 L at 12^th^ week, with the upper 95%CI margin arriving at the MID of 0.10 L. This effect size appears to be similar to that of the tiotropium plus formoterol combination, which yields an additional improvement of 0.11 L (95%CI: 0.07 L to 0.14 L) [34]. While preference for indacaterol over formoterol as an add-on to tiopiopium may improve adherence, comparative effectiveness of the two combinations on various secondary outcomes, as well as their safety, is uncertain [35]. Further head to head comparisons between the two combinations are needed to provide a firm basis for judgment.

Finally, for patients at high risk of exacerbation, currently the GOLD document [5] recommends the addition of inhaled corticosteroids on top of long acting bronchodilators. A network meta-analysis [36] demonstrated that indacaterol 75 ug has similar effect in improving FEV1 compared to formoterol plus budesonide or salmeterol plus fluticasone. Future head to head trials on these comparisons are needed to clarify the possible role of indacaterol in lowering the need of using inhaled corticosteroids amongst high risk patients. In addition, efficacy and safety of combining indacaterol and inhaled corticosteroids should be explored.

### Quality of the evidence and limitations of this review

Amongst 12 included trials, only seven clearly blinded both patients and investigators, and three blinded assessors. Given the subjective nature of all secondary outcomes included in this review (SGRQ, TDI, exacerbation, worsening of symptoms, and the dyspnoea component of BODE index), lack of blinding in these trials has led to a downgrade of quality of evidence for all secondary outcomes. Future trials would need to address this shortcoming, as well as providing detail descriptions on how random sequence generation and allocation concealment were achieved.

We have included all published trials on indacaterol and the comprehensiveness of search is confirmed by the manufacturer. Also, we were able to obtain unpublished data from the manufacturer. These data were presented as adjusted means instead of raw means. Nevertheless, we were informed that all means were adjusted for a same set of variables. Another limitation is that there are only a small number of trials in some of the subgroup analyses and hence the result may not be reliable.

### Agreements and disagreement with other studies or reviews

Our placebo controlled efficacy results are consistent with findings from two previous pooled analyses using a subset of RCTs included in this meta-analysis. One pooled analysis [37] of three RCTs [18], [25], [27] reported a FEV1 change of 0.16 L at 12^th^ week, which is identical to our synthesized results. Another pooled analysis [38] of three RCTs [23], [25], [27] reported a 6 month change of 1.01 and −4.4 respectively in TDI and SGRQ scoring at a indacaterol dosage of 150 ug. These effect sizes are similar to our findings. In a 1 year follow up [39] of patients who voluntarily maintain their use of indacaterol and placebo after the completion of Donohue et al.'s RCT [25], clinically relevant improvement in FEV1 and SGRQ were reported. After imputation, this study showed that Indacaterol 300 mg outperformed placebo in preventing exacerbations. Nevertheless, since less than half of the original participants joined the follow up, [39] strong impact from attrition bias has substantially lowered the trustworthiness of these findings.

Our findings are consistent with a previous network meta-analysis which reported similar efficacies between indacaterol and existing bronchodilators [40]. Compared to tiotropium, our results are concordant with a recent meta-analysis which has concluded that indacaterol fares marginally better in improving TDI and SGRQ [41]. In terms of safety, our results resonate with an existing review which highlighted low incidence of serious adverse events amongst indacaterol users [42].

### Novelty and limitation of this systematic review

While consistencies of results between our study and existing meta-analyses and narrative review [43] have strengthened our conclusions, it should be emphasized that in our subgroup meta-analysis has provided novel insight on the choice of indacaterol dosage. Using unpublished data from Novartis we have demonstrated that there is no significant improvement in efficacy when the dose is higher than 150 ug, implying that the use of lower dosage may be preferred as one death related to indacaterol use is found when the dose reaches 300 ug. This message has not been reported in previous reviews. Results related to dosage and death at 300 ug is tentative and further studies should be conducted to evaluate optimal indacaterol dosage.

In this systematic review, all included studies were initiated and sponsored by Novartis, and most of the studies were part of the application package for the regulatory authorities. Therefore, industry bias may lead to more favorable results and hence they should be interpreted conservatively [44]. In addition, we did not include any unpublished trial outcome data in our meta-analyses, and this may cause an inflation or deflation of efficacy and harm estimations. That said, it is unlikely that the exclusion of unpublished trials data would affect the statistical significance of a meta-analysis. Hence, the direction of effect reported in the present analysis should be considered as stable [45]. Finally, as Novartis is developing glycopyrronium/indacaterol combination therapy, future meta-analysis should consider trials evaluating this new treatment option [46].

## Conclusion

Indacaterol, at dosages of 75, 150 and 300 ug, provides clinically important improvement in FEV1, SGRQ, TDI and BODE index of patients with moderate to severe stable COPD by similar magnitude. In one trial, indacaterol was not found to be protective against exacerbation at 1 year even at its maximum EMA approved dose of 300 ug, and one treatment related death was reported at this dosage. Otherwise, its safety profile is good except for a higher rate of nasopharyngitis, which is a mild side effect. Efficacy of Indacaterol appears to be similar to all three commonly prescribed long-acting bronchodilators: salmeterol, formoterol and tiopropium. The addition of indacaterol on top of tiotropium yields clinically relevant, extra improvement on FEV1.

Future well-blinded RCTs are needed to investigate: (i) the comparative effectiveness of indacaterol at 150 ug, and the FDA approved dose of 75 ug; (ii) the efficacy of low dose indacaterol in preventing longer term exacerbations; and (iii) the potential add-on benefits of using indacaterol on top of tiotropium on the outcomes of SGRQ, TDI, exacerbation and BODE index.

## Supporting Information

File S1 Search strategy for MEDLINE.
(DOC)

File S2 PRISMA 2009 Checklist.
(DOC)
