# Supplementary material for: Indacaterol for Chronic Obstructive Pulmonary Disease: Systematic Review and Meta-Analysis
Source: PLoS One. 2013 Aug 14;8(8):e70784. doi: 10.1371/journal.pone.0070784 (PMC3743831; doi:10.1371/journal.pone.0070784)
Supplement: File S1 — Search strategy for MEDLINE. (DOC) [file pone.0070784.s001.doc]

**Table S1: Search strategy for MEDLINE**

Database: Ovid MEDLINE(R) 1946 to Present with Daily Update

Date: 30 Jan 2012

--------------------------------------------------------------------------------

1 Lung Diseases, Obstructive/ or exp Pulmonary Disease, Chronic Obstructive/ or emphysema$.mp. or (chronic$ adj3 bronchiti$).mp. or (obstruct$ adj3 (pulmonary or lung$ or airway$ or airflow$ or bronch$ or respirat$)).mp. or COPD.mp. or COAD.mp. or COBD.mp. or AECB.mp. (97803)

2 Indacaterol.tw,kf,nm,ot. (88)

3 Arcapta.tw,kf,nm,ot. (0)

4 Neohaler.tw,kf,nm,ot. (1)

5 Hirobriz Breezhaler.tw,kf,nm,ot. (0)

6 Onbrez Breezhaler.tw,kf,nm,ot. (1)

7 2 or 3 or 4 or 5 or 6 (89)

8 1 and 7 (58)

9 clinical trial.mp. or clinical trial.pt. or random:.mp. or tu.xs. (2837270)

10 8 and 9 (56)

11 limit 10 to humans (56)
